# Supplementary material for: Phylogenetic Analysis Based on DNA Barcoding and Genetic Diversity Assessment of Morinda officinalis How in Vietnam Inferred by Microsatellites
Source: Genes (Basel). 2022 Oct 25;13(11):1938. doi: 10.3390/genes13111938 (PMC9689669; doi:10.3390/genes13111938)
Supplement: Supplementary file 1 [file genes-13-01938-s001.zip › genes-1971236-supplementary.pdf]

Supporting information

Table S1. Geographical characteristics of the 37 samples of *M. officinalis* used in this study

**Samples**	**Pop.**	**Locations**	**Latitude (N)**	**Longitude (E)**
H1	Hue	Phong Xuan commune, Phong Dien district Hue province, Vietnam	75°05′38″	182°38′90″
H2		Phong Xuan commune, Phong Dien district Hue province, Vietnam	75°05′37″	182°38′90″
H3		Phong Xuan commune, Phong Dien district Hue province, Vietnam	75°05′33″	182°38′89″
H4		Phong Xuan commune, Phong Dien district Hue province, Vietnam	75°05′38″	182°38′81″
H5		Phong Xuan commune, Phong Dien district Hue province, Vietnam	75°05′38″	182°38′45″
H6		Phong Xuan commune, Phong Dien district Hue province, Vietnam	75°05′36″	182°38′47″
H7		Phong Xuan commune, Phong Dien district Hue province, Vietnam	75°05′64″	182°39′14″

H8	Phong Xuan commune, Phong Dien district Hue province, Vietnam	75°05′63″	182°39′22″
H9	Phong Xuan commune, Phong Dien district Hue province, Vietnam	75°05′66″	182°39′26″
H10	Phong Xuan commune, Phong Dien district Hue province, Vietnam	75°05′16″	182°38′05″
H11	Phong Xuan commune, Phong Dien district Hue province, Vietnam	75°05′11″	182°37′85″
H12	Phong Xuan commune, Phong Dien district Hue province, Vietnam	75°05′03″	182°37′87″
H13	Phong Xuan commune, Phong Dien district Hue province, Vietnam	75°04′88″	182°37′86″
H14	Phong Xuan commune, Phong Dien district Hue province, Vietnam	73°99′80″	181°60′86″
H15	Phong Xuan commune, Phong Dien district Hue province, Vietnam	74°00′58″	181°61′37″
H16	Phong Xuan commune, Phong Dien district Hue province, Vietnam	74°00′55″	181°61′36″

H17		Phong Xuan commune, Phong Dien district Hue province, Vietnam	74°00′86″	181°61′57″
H18		Phong Xuan commune, Phong Dien district Hue province, Vietnam	74°01′11″	181°62′06″
QN1	Quang Nam	Phuoc Son district, Quang Nam province, Vietnam	50°97′65″	170°16′74″
QN2		Phuoc Son district, Quang Nam province, Vietnam	50°97′64″	170°16′79″
QN3		Phuoc Son district, Quang Nam province, Vietnam	50°97′82″	170°16′92″
QN4		Phuoc Son district, Quang Nam province, Vietnam	50°99′56″	170°15′06″
QN5		Phuoc Son district, Quang Nam province, Vietnam	50°97′82″	170°16′92″
QN6		Phuoc Son district, Quang Nam province, Vietnam	50°97′95″	170°16′72″
QN7		Phuoc Son district, Quang Nam province, Vietnam	50°97′95″	170°16′72″
QN8		Phuoc Son district, Quang Nam province, Vietnam	50°99′55″	170°15′00″
QN9		Phuoc Son district, Quang Nam province, Vietnam	50°99′56″	170°14′95″

QN10		Phuoc Son district, Quang Nam province, Vietnam	50°99′55″	170°15′00″
QB1	Quang Binh	Minh Hoa district, Quang Binh Province, Vietnam	17°45'14.94	106° 4'6.04
QB2		Minh Hoa district, Quang Binh Province, Vietnam	17°45'9.23	106° 4'7.34
QB3		Minh Hoa district, Quang Binh Province, Vietnam	17°45'7.63	106°4'12.89
QB4		Minh Hoa district, Quang Binh Province, Vietnam	17°45'8.88	106° 4'16.14
QB5		Minh Hoa district, Quang Binh Province, Vietnam	17°45'5.60	106° 4'17.81
QB6		Minh Hoa district, Quang Binh Province, Vietnam	17°45'4.06	106° 4'20.23
QB7		Minh Hoa district, Quang Binh Province, Vietnam	17°45'4.49	106° 4'21.94
QB8		Minh Hoa district, Quang Binh Province, Vietnam	17°44'59.83	106° 4'30.69
QB9		Minh Hoa district, Quang Binh Province, Vietnam	17°44'57.64	106° 4'30.42

Table S2. The primers used for PCR and sequencing in this study

SSR loci	Forward sequences	Reverse sequences	Repeat motif	Expected allele size (bp)	Annealing temperature (ºC)	Genbank accession no.	Ref.

MO02	GATTAACGCCACTGTTG	CGGATACGGAGGAGGTTA	(CCG)5...(CCG)6	204	50	MF496207	
MO04	TCTCTTGCCTCTGGTAGTT	ACCCTCAATGGAGAACAAC	(GCC)6…(TGC)5	164	51	MF496208	
MO05	TAACTAATGGCGTACTTGG	GCTCATCTACCACTACTGAA	(GAA)7(AGA)5	214	50	MF496209	
MO12	TTGGCTGTGTGCTTCTTT	ATTCCTTCCTTCCTCCTAATC	GT)9	135	50	MF496210	
MO19	TCTCGCATTCAGGCAAAG	TCTCGCATTAGGCAAAG	(GA)8	111	51	MF496211	
MO26	TAGTTGAGCCGCTTGAGT	GGTTCCATTCCATTCAGAGG	(CT)7	193	51	MF496212	
MO30	CATGAGTTGCAGATGGAAT	AGAGACAGAGATTAGACGAA	(AT)7	158	51	MF496213	[16]
MO38	AAAGTGGGTGAGGGTTAGA	CAGAGTGGTGGACGAATAG	(AG)6	206	52	MF496216	
MO39	TAGACCATAGGCTGGAGTT	TTGCTAAGGAATCAGGAGTT	(TG)6	182	51	MF496217	
MO41	CACACTATACTCAAGCACATC	GTTGGGCTGACCTTTCCTT	(TA)6	141	50	MF496218	
MO43	CTCTCCTTCTCCTTATCTCTG	GGAACTGGCAATGGACTT	(CT)6	261	50	MF496219	
MO47	CTGCGGAGTGCATAAGAA	TAGCCAATCGTAGAGAATAG	(GA)6	173	50	MF496220	
MO53	TCCACAGGCTAAGATTACAC	CCAGCATAGTCTTCCTCTA	(CT)6	116	50	MF496221	

		G				
MO57	GCATCATTAGAGCTACTAGAC	TGTTGTCACTGTACTTCATC	(CAG)8	172	50	MF496222
MO60	TGAATTGGGTGAAGAACCA	CTTAACTCACTGATCTGTCTG	(CAA)8	154	50	MF496223
MO61	TCAAGGACAGTATTGTGGAA	CATCATCATTGCTGCTCTTA	(TAA)8	189	50	MF496224
MO63	ACATTGCCGAATACCATCT	CCAGAGTTGTTGTCAAGTT	(GTG)7	134	50	MF496225
MO88	TCCGACTTGCTTCATTGG	TTGGGCTCAACCTTCTCA	(TTG)6	197	51	MF496226
MO89	AGCCGATACTAAACTGTCAA	AACCATCACTCAATGTTCCA	(ATC)6	240	51	MF496227
MO90	TTACAACTGTGGCAGAACT	ATCCAGCACTACCAATCC	(GTG)6	170	50	MF496228
MO94	ACTAAGCCGAGTGAATTACA	TTCCAACCTGCCTATCCA	(TGC)6	201	50	MF496229
MO96	AGGTAACTTCAGTCAACAC	TGGGAATGTCAACAGAAATC	(CAA)6	122	50	MF496230

Table S3. The samples were deposited in the GenBank and their accession numbers

No.	Samples	Primer	Population	Accession
1	H01	ITS1	TTH	ON819584.1
2	H02	ITS1	TTH	ON819586.1
3	H03	ITS1	TTH	ON819588.1
4	QN01	ITS1	QN	ON819590.1
5	QN02	ITS1	QN	ON819591.1
6	QN03	ITS1	QN	ON819592.1
7	QB01	ITS1	QB	ON819596.1

8	QB02	ITS1	QB	ON819597.1
9	QB03	ITS1	QB	ON819598.1
10	H01	ITS2	TTH	ON819585.1
11	H02	ITS2	TTH	ON819587.1
12	H03	ITS2	TTH	ON819589.1
13	QN01	ITS2	QN	ON819593.1
14	QN02	ITS2	QN	ON819594.1
15	QN03	ITS2	QN	ON819595.1
16	QB01	ITS2	QB	ON819599.1
17	QB02	ITS2	QB	ON819600.1
18	QB03	ITS2	QB	ON819601.1
19	H01	MatK	TTH	ON926564.1
20	QN01	MatK	QN	ON926565.1
21	QN02	MatK	QN	ON926566.1
22	QN03	MatK	QN	ON926567.1
23	H01	rbcL	TTH	ON926568.1
24	H02	rbcL	TTH	ON926569.1
25	QN01	rbcL	QN	ON926570.1
26	QN03	rbcL	QN	ON926571.1
27	QB02	rbcL	QB	ON926572.1
